# Theory of Mind and Its Neuropsychological and Quality of Life Correlates in the Early Stages of Amyotrophic Lateral Sclerosis

**DOI:** 10.3389/fpsyg.2016.01934

**Published:** 2016-12-12

**Authors:** Francesca Trojsi, Mattia Siciliano, Antonio Russo, Carla Passaniti, Cinzia Femiano, Teresa Ferrantino, Stefania De Liguoro, Luigi Lavorgna, Maria R. Monsurrò, Gioacchino Tedeschi, Gabriella Santangelo

**Affiliations:** ^1^Department of Medical, Surgical, Neurological, Metabolic and Aging Sciences – MRI Research Center SUN-FISM, Università degli Studi della Campania “L. Vanvitelli”Naples, Italy; ^2^Department of Psychology, Università degli Studi della Campania “L. Vanvitelli”Caserta, Italy

**Keywords:** amyotrophic lateral sclerosis, theory of mind, social cognition, emotion attribution, quality of life

## Abstract

This study aims to explore the potential impairment of Theory of Mind (ToM; i.e., the ability to represent cognitive and affective mental states to both self and others) and the clinical, neuropsychological and Quality of Life (QoL) correlates of these cognitive abnormalities in the early stages of amyotrophic lateral sclerosis (ALS), a multisystem neurodegenerative disease recently recognized as a part of the same clinical and pathological spectrum of frontotemporal lobar degeneration. Twenty-two consecutive, cognitively intact ALS patients, and 15 healthy controls, underwent assessment of executive, verbal comprehension, visuospatial, behavioral, and QoL measures, as well as of the ToM abilities by Emotion Attribution Task (EAT), Advanced Test of ToM (ATT), and Eyes Task (ET). ALS patients obtained significantly lower scores than controls on EAT and ET. No significant difference was found between the two groups on ATT. As regard to type of ALS onset, patients with bulbar onset performed worse than those with spinal onset on ET. Correlation analysis revealed that EAT and ET were positively correlated with education, memory prose, visuo-spatial performances, and “Mental Health” scores among QoL items. Our results suggest that not only “cognitive” but also “affective” subcomponents of ToM may be impaired in the early stages of ALS, with significant linkage to disease onset and dysfunctions of less executively demanding conditions, causing potential impact on patients’ “Mental Health.”

## Introduction

Theory of Mind (ToM), regarded as an essential prerequisite for successful human social interaction (Adolphs, 2003), is the ability to infer and predict intentions, thoughts, desires and behavioral reactions to oneself and others, through an awareness that others have a mind with “affective” and “cognitive” mental states that may differ from one’s own (Frith and Frith, 1999). Considering that social interaction, among the cognitive aspects that may impact adherence to treatment and patients’ quality of life (QoL), has been increasingly shown to be impaired in neurodegenerative diseases, including amyotrophic lateral sclerosis (ALS; Elamin et al., 2012; Santangelo et al., 2012; Yamada et al., 2015; Burke et al., 2016a), an early assessment of this domain in the disease course could be useful for positively conditioning patients’ management and prognosis. In particular, with regard to ALS, a multisystem neurodegenerative disease, not only characterized by motor dysfunctions, but also by extra-motor symptoms, belonging to the clinical and pathological spectrum of FTLD (Lillo et al., 2012; Ling et al., 2013; Trojsi et al., 2015; Burrell et al., 2016), the growing evidence of social cognition impairments may have important implications on patient’s QoL and ability to engage competently in end-of-life decisions (Chiò et al., 2004; Lulé et al., 2013; Körner et al., 2015).

From the anatomical point of view, the cognitive and affective aspects of ToM have been demonstrated to be supported by dissociable, yet interacting, prefrontal networks (Shamay-Tsoory et al., 2004). Specifically, the “cognitive” ToM network primarily engages the dorsomedial prefrontal cortex, the dorsal anterior cingulate cortex and the dorsal striatum, while the “affective” ToM network engages the ventromedial and orbitofrontal cortices, the ventral anterior cingulate cortex, the amygdala and the ventral striatum (Poletti et al., 2012). In this regard, several lesion studies have provided evidence that ToM could be considered a multidimensional construct (Shamay-Tsoory et al., 2005, 2006; Gupta et al., 2012; Sebastian et al., 2012) and the different ToM subcomponents have been explored *in vivo* by several cognitive and affective ToM tasks, such as the false-beliefs tasks (Wimmer and Perner, 1983; Baron-Cohen et al., 1985) and the Reading the Mind in the Eyes or Eyes Task (ET; Baron-Cohen et al., 1997, 2001), the former prototypical for the assessment of cognitive ToM, the latter for the assessment of the affective ToM. However, there is a body of literature criticizing how ToM is used and investigated (Frith and Happé, 1994a; Bloom and German, 2000).

With regard to the investigation of “affetive” and “cognitive” components of ToM in neurologic disorders, significant deficits have been revealed in cortical (i.e., Alzheimer’s disease and Frontotemporal Lobar Degeneration or FTLD; Poletti et al., 2012) and frontal-subcortical (Snowden et al., 2003; Meier et al., 2010; Crespi et al., 2014) neurodegenerative diseases. Impairment of social cognition has also been described in several cohorts of patients in heterogeneous stages of ALS (Girardi et al., 2011; Van der Hulst et al., 2015; Burke et al., 2016a,b). Moreover, abnormalities of both cognitive and affective ToM, related to social behavior dysfunctions, such as loss of empathy and apathy (Gregory et al., 2002; Girardi et al., 2011), have been recently confirmed as a prominent feature of behavioral profile in advanced ALS (Van der Hulst et al., 2015). Approximately 5–15% of ALS patients meet the diagnostic criteria for FTLD (Neary et al., 1998; Rascovsky et al., 2011), predominantly developing clinical features typical of the behavioral variant frontotemporal dementia (bvFTD; Lomen-Hoerth, 2004) and a larger proportion of non-demented ALS patients shows cognitive (mainly involving the executive domain) and/or behavioral deficits, belonging to a spectrum of symptoms frequently recognized within FTLD syndromes (Lomen-Hoerth, 2004; Abrahams et al., 2005; Burrell et al., 2016). With regard to social cognitive deficit in ALS, as well as in overall the disease spectrum of FTLD (Elamin et al., 2012), Burke et al. (2016b) demonstrated that ET is a valid measure of the cognitive decline in this domain in ALS patients, also in terms of severity, showing, in a previous study (Burke et al., 2016a), that patients with ALS with bulbar onset (ALS-B) may perform worse in this task, thus supporting the view that ALS-B may represent a biologically more aggressive phenotype of the disease (Cardenas-Blanco et al., 2014). Moreover, Van der Hulst et al. (2015) revealed that in advanced ALS impairment of both subcomponents of ToM may be associated with apathy and impairment of frontotemporal-related abilities, such as verbal fluency and naming abilities, suggesting that evidence of both deficits may reflect a widespread degeneration of frontal lobes as the disease progresses.

To note, there are opposing views in the literature with regard to the relationship between ToM performances and executive or behavioral skills (Baron-Cohen et al., 1985; Bertoux et al., 2016) and, with regard to ALS, some studies, which were performed in small and phenotypically heterogeneous samples of patients, showed that ToM deficits were not selectively associated with executive dysfunctions (Meier et al., 2010; Cavallo et al., 2011; Girardi et al., 2011). On the contrary, Carluer et al. (2015) revealed a significant association between alterations of the cognitive subcomponent of ToM and executive functions in the early stages of the disease.

On this background, given that the potential existence of ToM abnormalities in the early stages of ALS and in different ALS onsets are not still completely elucidated, in the present study, we aimed to investigate whether cognitive and affective ToM are impaired in a small sample of well-characterized ALS patients, who were in King’s stages 1 and 2 of the disease (Balendra et al., 2015) and were stratified for disease onset. We classified the patients according to the King’s staging system, that account for having reached different milestones of the disease related to the number of regions involved (Balendra et al., 2015), differently from Milano-Torino Staging system (MITOS; Chiò et al., 2015), that captures the observed progressive loss of independence and function. Moreover, considering that correlations between ToM performances and neurobehavioral and QoL aspects, although remarkable for ALS patients’ management, are still largely debated, we also explored neuropsychological and behavioral correlates of cognitive and affective ToM performances in our population, assessing potential impairment of executive, memory [i.e., the two cognitive domains reported to be impaired in ALS (Phukan et al., 2012; Montuschi et al., 2015)] and behavioral domains, arising from alteration of frontal and subcortical regions (Tsermentseli et al., 2012), and the impact of ToM dysfunctions on patients’ QoL.

## Materials and Methods

### Patients

In the present study, 22 right-handed and native Italian speakers patients, with probable and probable laboratory- supported ALS, according to the El-Escorial revised criteria (Brooks et al., 2000), were consecutively recruited at the First Division of Neurology of the Università degli Studi della Campania “L. Vanvitelli” (Naples, Italy). To be included in this study, which aimed to evaluate ToM performance in ALS patients without the confounding effect of other cognitive abnormalities, patients had to satisfy the following inclusion criteria: (a) to have an age- and education-adjusted score >71.78 on Addenbroke’s Cognitive Examination Revised (ACE-R) [according to the Italian validated version of ACE-R (Siciliano et al., 2016)], a tool used to screen participants in terms of overall cognitive ability; (b) to exhibit preserved verbal comprehension abilities, needed to correctly execute the ToM tasks adopted in this study, assessed by Token Test, according to previous evidence (Hermann et al., 1992; Santangelo et al., 2012; Maseda et al., 2014) [age- and education-adjusted score >26.25 (Spinnler and Tognoni, 1987)]; (c) to be in stages 1 and 2 of ALS according to the King’s clinical staging system, based on the appearance of sequential clinical milestones during the ALS course, without including cognitive information (i.e., stage 1 = impairment of one body site; stage 2 = impairment of two body sites; stage 3 = impairment of three body sites; stage 4 = non-invasive ventilation or percutaneous endoscopic gastrostomy; Balendra et al., 2015); (d) to use no medication influencing cognitive performances. Clinical parameters were measured in all ALS patients using the ALSFRS-R score, an ALS-specific measure of functional ability (Cedarbaum et al., 1999), and the upper motor neuron (UMN) score, a measure of pyramidal dysfunction through the evaluation of the number of pathological reflexes elicited from 15 body sites (Turner et al., 2004).

None of patients carried C9ORF72, SOD1, TARDBP, and FUS/TLS genes mutation.

Fifteen healthy control subjects (HCs), enrolled by “word of mouth,” were age-, sex-, and education-matched with ALS patients for adequately selecting the control group. Given that HCs underwent the same neuropsychological assessment of ALS patients, the recruited HCs were cognitively normal and those with comorbid neurological, psychiatric, or medical conditions that could affect cognition were excluded.

The research was conducted according to the principles expressed in the Declaration of Helsinki. Ethics approval was obtained from the Ethics Committee of the Università degli studi della Campania “L. Vanvitelli”. Written informed consent was obtained from each participant.

### Neuropsychological Assessment

A 60-min neuropsychological battery, assessing cognitive functioning/ability (global cognitive assessment and executive, memory and abstract reasoning abilities), ToM abilities, frontal behavioral disorders, and QoL, was designed by a team of neurologists and neuropsychologists experienced in the study and management of motor neuron diseases and cognitive decline. Although this neuropsychological battery was not ALS-specific, the tests administered have been widely used in ALS and other movement disorders (Phukan et al., 2012; Santangelo et al., 2012; Montuschi et al., 2015; Burke et al., 2016a), since they were less influenced by motor symptoms. All tests were administered in the morning following the same sequence for avoiding possible interference of the answers of one test over the others (Montuschi et al., 2015). In particular, after 1 day from assessment of global cognitive functions and, then, executive, memory, visuospatial, behavioral, and mood functions, patients underwent ToM assessment. Moreover, if the subject was too tired during testing, a further session was scheduled to complete the battery within 2 weeks after the first one. Considering that respiratory dysfunction may impact cognitive performances (Kim et al., 2007; Gülhan et al., 2015; Braley et al., 2016), oxygen saturation, and forced vital capacity (FVC) were measured at the time of each examination (i.e., none participant showed oxygen saturation <92 mmHg and FVC < 80%; Montuschi et al., 2015).

Given the aim of exploring neuropsychological and behavioral correlates of cognitive and affective ToM performances in our population, the neuropsychological protocol adopted allowed to assess: (1) executive performances (i.e., mainly the inhibitory control as specific executive function) by Stroop Color-Word Interference test using the Stroop Executive Factor (SEF; Caffarra et al., 2002), that accounts for motor disability (Phukan et al., 2012; Burke et al., 2016a); (2) long-term verbal memory by memory prose test (Novelli et al., 1986); (3) non-verbal abstract reasoning and current intellectual functioning by Raven’s Colored Progressive Matrices (RCPM; Carlesimo et al., 1996); and (4) behavioral dysfunctions and mood by Frontal Systems Behaviour (FrSBe) Scale (Grace et al., 1999), referring to the total scores at the time of examination, derived from the caregivers’ forms, proven to be not influenced from motor symptoms (Terada et al., 2011; Chiò et al., 2012) and Beck Depression Inventory-II (BDI-II; Innamorati et al., 2013). Caregiver burden was assessed by means of Caregiver Burden Inventory (CBI), a self-administered questionnaire evaluating the effect of caring on the caregiver (Novak and Guest, 1989). It comprised 24-item and its scores ranged from 0 (lowest level) to 100 (highest level). Finally, the ALS patients completed the Italian version of the Short Form-36 (SF-36; Apolone and Mosconi, 1998), in order to evaluate eight domains of QoL (i.e., physical functioning, limitations in everyday activities, bodily pain, general health, vitality, social functioning, emotional problems, and mental health). For each domain an aggregate percentage score was produced, which ranged from 0% (lowest or worst possible level of functioning) to 100% (highest or best possible level of functioning). In the present study, we considered the Italian standards to calculate *z*-score for each domain of SF-36 (Apolone and Mosconi, 1998).

After this preliminary evaluation, to explore ToM abnormalities in the early stages of ALS and in different ALS onsets, the participants underwent three ToM tasks that covered both cognitive and affective subcomponents. In particular, the advanced test of ToM (ATT), mainly considered a cognitive task, has been designed to investigate the ability to attribute mental states to others (Happé, 1994; Prior et al., 2003). The task included 13 written stories describing naturalistic situations in which two or more characters interacted with each other as in familiar or social contexts (e.g., children arguing about a toy’s property, a mother chiding a son for not appreciating food, children pretending to behave as adults). The examiner had to read each story and, then, asked the participants why the characters behaved as they did. The total score ranged from 0 (worst performance) to 13 (best performance). Successful performance requires attribution of mental states such as desires, beliefs or intentions, and also higher orders mental states such as one character’s belief about what another character knows. The emotion attribution task (EAT; Prior et al., 2003), used to assess the ability to attribute emotional states to others, included 35 written stories describing emotional situations (e.g., an employee apprehending to receive an extra salary, a man attacked by a big black spider, a woman finding a worm in her food): the examiner had to read each story and, then, asked the participants what the main protagonists might feel in that situation. Five stories were designed to elicit attribution of sadness, fear, embarrassment, disgust, happiness, anger, or envy (five stories for each emotion). The total score ranges from 0 (worst performance) to 35 (best performance). Finally, we also administered the ET, consisting of the presentation of photographs of the eye regions of human faces to participants who were required to choose which word best describes what the individual in the photograph is thinking or feeling (Baron-Cohen et al., 2001). A control task, designed to investigate participants’ ability to correctly identify human physical attributes, such as gender, was undertaken subsequently. Participants provided verbal responses and could take as long as they wanted to respond. All participants were encouraged to consult a glossary of all mental state terms for a correct interpretation of the lexicon in any case where they were unsure of a word (Baron-Cohen et al., 2001). To note, with regard to HCs, the ATT and ET scores were similar to those revealed in other samples of normal adults (Baron-Cohen et al., 2001; Santangelo et al., 2012, 2013; Sato et al., 2016).

### Statistical Analysis

With regard to continuous variables, a multivariate analysis of variance (MANOVA), followed by multivariate analysis of covariance (MANCOVA), where appropriate, were performed for between-group comparisons. To reduce the risk of type I error associated with multiple comparisons, Bonferroni adjustments were made, with *p* set at α/*n* (where *n* = number of variables considered: 0.05/8 = 0.006).

To compare ALS patients’ performance on cognitive and affective ToM tasks, standardized *z* scores were calculated with reference to control group’s means and SD. The *z* scores displayed the relative degree of impairment from normal performance in SD units, thereby allowing to compare scores on ET, ATT and EAT within ALS group by a one-way ANOVA for repeated measures. We checked the data for normality using several procedures (i.e., Shapiro–Wilk normality test, kurtosis and skewness). Within the ALS group, Pearson’s correlation analysis, followed by Bonferroni correction (0.05/5 = 0.01 for demographic and clinical variables; 0.05/5 = 0.01 for neuropsychological variables; 0.05/14 = 0.003 for neurobehavioral variables) was carried out to explore potential associations between ToM performances and demographic (age and education), clinical (disease duration, and disease severity assessed by ALSFRS-R and UMN), cognitive, behavioral, and QoL features.

## Results

### Demographic Variables

Twenty-two (9 females and 13 males) ALS patients (i.e., 9 with bulbar-onset and 13 with spinal-onset ALS) and 15 HCs (7 females and 8 males), age- and education-matched, were included in the present study. With regard to characterization of clinical phenotypes of ALS (Chiò et al., 2011), patients exhibited “classic” (Charcot’s; i.e., onset of symptoms in the upper/lower limbs, with clear but not predominant pyramidal signs) and “bulbar” (i.e., bulbar onset without peripheral spinal involvement for the first 6 months after symptoms onset) phenotypes. Moreover, according to the King’s clinical staging system (Balendra et al., 2015), based on the identification of sequential “clinical milestones” during the ALS course, 10 patients were in stage 1 and 12 in stage 2. For more details about demographic and clinical characteristics, see **Table 1**.

**Table 1:** Demographic, clinical, and neuropsychological features of ALS patients and healthy control subjects (HCs; mean ± SD).

Demographic and clinical parameters	ALS patients (*n* = 22)	HCs (*n* = 15)	*F*/χ^2^	*p*
Age	58.19 ± 9.63	55.4 ± 8.72	1.586	0.218
Education (years)	11.38 ± 4.55	9.93 ± 2.68	2.584	0.119
Gender (M:F)	13:9	8:7	0.120	0.729
Disease duration^∗^	18.73 ± 10.87	–		
ALSFRS-R total score	41 ± 3.86	–		
ALSFRS-R bulbar subscore	10.45 ± 1.68	–		
ALSFRS-R arm subscore	9.54 ± 2.1	–		
ALSFRS-R leg subscore	9.18 ± 2.46	–		
ALSFRS-R respiratory subscore	11.7 ± 0.7	–		
UMN score	6.50 ± 4.68	–		


*^∗^Months from symptom onset to assessment. ALSFRS-R, amyotrophic lateral sclerosis functional rating scale; UMN, upper motor neuron.*

### Neuropsychological Assessment: Between-Group Comparisons

With regard to neuropsychological and neurobehavioral variables, MANOVA with Bonferroni *post hoc* test showed significant difference between ALS patients and HCs on memory prose test (*p* < 0.006; **Table 2**). Therefore, MANCOVA analysis, with memory prose test as covariate, showed that ALS patients performed significant lower scores than HCs on EAT and ET, but not on ATT task (**Table 3**). The values of η^2^ (**Table 3**) for each ToM variable were >0.14, indicating a large effect (Cohen, 1988). With regard to the within-group comparisons, no significant differences were revealed by comparing EAT, ATT, and ET scores in the ALS group (i.e., one-way ANOVA for repeated measures: *F* = 1.217, *p* = 0.301).

**Table 2:** Neuropsychological features of ALS patients and HCs (mean ± SD).

Neuropsychological parameters [raw scores]	ALS patients (*n* = 22)	HCs (*n* = 15)	*F*	*p*
ACE-R	87.71 ± 8.96	93.07 ± 3.24	4.522	0.042
Token test	33.72 ± 2.02	34.93 ± 1.38	3.429	0.075
Memory prose test	11.53 ± 2.83	15.13 ± 1.60	14.476	**0.001**
RCPM	26.15 ± 5.19	29.80 ± 4.52	5.554	0.026
Stroop executive factor	0.30 ± 0.733	0.20 ± 0.561	0.600	0.445
BDI	12.37 ± 6.57	10.40 ± 8.24	0.640	0.430
CBI	19.28 ± 15.74	–		
SF-36: physical functioning (*z* score)	43.33 ± 27.23 (-1.7)	–		
SF-36: role-physical (*z* score)	28.33 ± 39.94 (-1.3)	–		
SF-36: bodily pain (*z* score)	57.73 ± 24.98 (-0.5)	–		
SF-36: general health (*z* score)	43.13 ± 17.18 (-0.9)	–		
SF-36: vitality (*z* score)	54 ± 20.80 (-0.3)	–		
SF-36: social functioning (*z* score)	60.60 ± 23.06 (-0.7)	–		
SF-36: role-emotional (*z* score)	50.87 ± 39.53 (-0.6)	–		
SF-36: mental health (*z* score)	62.40 ± 20.2 (-0.2)	–		
FrSBe [caregiver forms, total score]	79.33 ± 12.12	–		
FrSBe [caregiver forms, apathy subscore]	25.77 ± 5.5	–		
FrSBe [caregiver forms, disinhibition subscore]	22.05 ± 3.81	–		
FrSBe [caregiver forms, executive dysfunction subscore]	31.5 ± 6.1	–		


*ACE-R, Addenbrooke’s cognitive examination; CBI, caregiver burden inventory; BDI, beck depression inventory; FrSBe, frontal systems behaviour; RCPM, Raven’s colored progressive matrices; SF-36, short form-36. The *z* score for each domain of SF-36 was calculated using mean and SD of Italian normative sample (Apolone and Mosconi, 1998). The statistically significant results after Bonferroni correction [*p* < 0.006] are reported in bold.*

**Table 3:** Performance of ALS patients and control subjects on tasks assessing ToM abilities (mean ± SD).

Parameters	ALS patients	Controls	*F*	*P*	η^2^
	(*n* = 22)	(*n* = 15)			
EAT	24.95 ± 3.72	27.47 ± 3.46	8.606	**0.001**	0.381
ATT	8.14 ± 2.57	8.67 ± 1.40	1.514	0.237	0.098
ET	20.67 ± 6.02	22.93 ± 5.12	6.878	**0.004**	0.329


*ATT, advanced test of ToM; EAT, emotion attribution task; ET, eyes test; ToM, theory of mind.*

*The statistically significant results are reported in bold.*

With regard to phenotypic characterization, statistical analysis showed that patients with bulbar-onset had lower scores than patients with spinal-onset on ET, while no significant differences between the two groups were found on ATT and EAT (**Table 4**). Moreover, within both bulbar- and spinal-onset groups, EAT, ATT, and ET scores were not significantly different (by one-way ANOVA for repeated measures: in bulbar-onset group, *F* = 0.605, *p* = 0.485; in spinal-group onset, *F* = 2.022, *p* = 0.156).

**Table 4:** Neuropsychological performances (mean ± SD) of ALS patients with bulbar-onset compared to those of patients with spinal-onset.

Neuropsychological parameters (raw scores)	Bulbar-onset ALS	Spinal-onset ALS	*F*	*p*
ACE-R	87 ± 9.84	89.45 ± 8.07	0.045	0.834
Token test	33.20 ± 2.16	34.32 ± 1.87	0.725	0.406
Memory prose test	11.50 ± 3	12.36 ± 2.22	0.689	0.417
RCPM	25.40 ± 6.10	27 ± 5.27	0.641	0.434
Stroop executive factor	0.20 ± 0.477	0.36 ± 0.924	0.059	0.811
EAT	24 ± 4.69	25.82 ± 3.28	0.643	0.433
ATT	7.80 ± 2.77	8.82 ± 2.08	0.812	0.379
ET	16 ± 5.74	23 ± 5.58	0.438	**0.033**


*ACE-R, Addenbrooke’s cognitive examination; ATT, advanced test of theory of mind; CBI, caregiver burden inventory; EAT, emotion attribution task; ET, eyes test; FrSBe, frontal systems behavior scale; RCPM, Raven’s colored progressive matrices; SF-36, short form-36.*

*The statistically significant results are reported in bold.*

### Correlation Analysis

Within the ALS sample, EAT and ET scores were significantly correlated with each other (*r* = 0.586, *p* = 0.011), while ATT did not correlate with other ToM scores. EAT scores were significantly correlated with education (*r* = 0.587, *p* = 0.006), ACE-R (*r* = 0.593, *p* = 0.008), memory prose (*r* = 0.659, *p* = 0.003), RCPM (*r* = 0.655, *p* = 0.003), and SF-36 “Mental Health” (*r* = 0.570, *p* = 0.033; uncorrected level of significance) scores, while ET scores were correlated with ACE-R (*r* = 0.581, *p* = 0.014; uncorrected level of significance), memory prose (*r* = 0.546, *p* = 0.029; uncorrected level of significance) and RCPM (*r* = 0.510, *p* = 0.044; uncorrected level of significance) scores. Finally, EAT, ET, and ATT scores were not correlated with other executive and non-executive neuropsychological variables and behavioral measures were not associated with any ToM scores.

## Discussion

The present cross-sectional study revealed an early impairment of ToM abilities in ALS patients, especially in those with bulbar onset, thus suggesting that non-demented ALS patients may have difficulties in attributing emotions and mental states to others. Moreover, from early stages of ALS, this neuropsychological profile may be significantly related to altered memory prose and non-verbal abstract reasoning. Another evidence derives from the association between impaired performances on EAT and its impact on health-related QoL of ALS patients, considering the potential consequences of deficits of social conduct, linked to ToM alterations, on QoL.

Abnormalities of the attribution of emotional and social intentions, found in several cohorts of ALS patients (Cavallo et al., 2011; Girardi et al., 2011), resemble the affective profile that characterizes FTLD patients, especially in mild and moderate stages of bvFTD (Torralva et al., 2015), corroborating the theory of a clinicopathological continuum between ALS and FTLD (Lillo et al., 2012; Ling et al., 2013; Trojsi et al., 2015). In particular, patients with bvFTD are characterized, from the early stages of disease, by significant changes in personality and social conduct (Piguet et al., 2011), mainly related to early alterations of ventromedial prefrontal areas (Lillo et al., 2012), where a similar pattern of prefrontal structural (Lillo et al., 2012; Crespi et al., 2014) and functional (Trojsi et al., 2015) abnormalities may also be revealed in ALS patients, although in later stages of the disease. Our findings remark that an impairment of both subcomponents of ToM, especially of the affective one, explored by both EAT and ET, might be considered as an extra-motor symptom occurring since the early stages of ALS, allowing to speculate that these poor performances may reflect mainly an early medial and orbital prefrontal cortex dysfunction, as also shown in early clinical stages of frontal lobe degeneration in both ALS (Van der Hulst et al., 2015) and bvFTD (Torralva et al., 2015). However, despite the large amount of literature investigating the behavioral and neural bases of mentalizing abilities in neurological conditions, there is still a lack of validated neuropsychological tools specifically designed to assess each ToM subcomponent (Dodich et al., 2015; Schaafsma et al., 2015). To note, among the experimental approaches used in neurological disorders, not all investigations on ALS aimed to assess both cognitive and affective subcomponents of ToM (Girardi et al., 2011; Carluer et al., 2015) and some authors explored mainly the affective subcomponent using non-verbal tasks, based on visual emotion recognition (such as ET or preference judgment task or false-belief task; Cavallo et al., 2011; Girardi et al., 2011; Crespi et al., 2014; Carluer et al., 2015; Van der Hulst et al., 2015; Burke et al., 2016a).

The distinction between affective and cognitive subcomponents of ToM has been essentially demonstrated by using different ToM tests with respect to their cognitive (e.g., belief about belief) or affective (e.g., belief about feelings) request. In this regard, a sound tool to investigate both ToM subcomponents using a verbal task is the “faux pas recognition” (FPR) test in which the participants hear 10 stories read aloud, containing a social faux pas and 10 control stories. After each story, participants are asked whether anyone said anything that they should not have said (i.e., evaluation of the affective subcomponent) and, when a faux pas is detected, further clarifying questions are proposed in order to evaluate the understanding of the mental states of the agents involved in the stories (i.e., evaluation of the cognitive component). In our study a strength point of the experimental design was to have investigated both subcomponents using verbal and non-verbal tasks in order to adequately assess the multifaceted aspects of the ToM abilities. However, there are some intrinsic limitations of the ToM tasks that, we adopted. In fact, there are no specific considerations concerning the role of ATT in the affective rather than in the cognitive ToM (Happé, 1994): AAT could be considered mainly a cognitive task, although it seems to be less associated to executive functions and less sensitive to neurological disorders (Aboulafia-Brakha et al., 2011). Moreover, it is to take into account that personality traits, especially with regard to antisocial component and self-presentation (i.e., the tendency to report social desirability), may impact Happé’s test of ToM also in non-psychopathic subjects (Nentjes et al., 2015), probably influencing ToM performances also in HCs subjects.

With regard to ET, considered the prototypical test to explore the affective subcomponent, it has been proven that cognitive ToM and executive abilities may impact the performance on this test (Bull et al., 2008; Dal Monte et al., 2014) that, therefore, should not be considered as a purely affective task.

On this background, our correlation analysis between the ToM scores and those derived from memory prose and RCPM tests showed that these neuropsychological measures were related to both EAT and ET, although only correlations with EAT were significant after Bonferroni correction. Together, these results support the hypothesis that mechanisms involving not only executively demanding abilities may be linked to early defects of ToM in ALS. To note, with regard to the debated association existing between executive and ToM domains, Bertoux et al. (2016), by applying a “clustering” approach to a large neuropsychological database derived from a population of bvFTD patients, revealed that executive functions “clustered” separately from ToM measures, substantially demonstrating that they were distinct components. Interestingly, Bertoux et al. (2016) found different relationships with executive functions across ToM subcomponents, showing that only the intention and empathy components, as measured by the FPR test, were linked to attention/working memory and verbal abstraction performances. Moreover, the lack of significant association between executive performances (especially the inhibitory control) and ToM performances may be explained considering that the advanced ToM tasks adopted have been proven to be more associated to language and memory functions in other neurological disorders (Castelli et al., 2011; Freed et al., 2015; Robinson et al., 2016), as also shown in our study with regard to the association between EAT and memory prose performances.

Our observation of a significant association between dysfunctions of less executively demanding conditions (i.e., memory and RCPM performances) and impairment of ToM in early ALS may suggest a potential independence between executive abilities and ToM performances. Some evidence supports a stronger association between executive functions and “fluid intelligence,” both involving mainly dorsolateral prefrontal networks (Roca et al., 2012), in contrast to ToM processes, hypothesized to be probably more dependent from medial prefrontal networks, especially with regard to the affective subcomponent (Shamay-Tsoory et al., 2005, 2006; Xi et al., 2011; Bertoux et al., 2016). However, more recently, Burke et al. (2016b) demonstrated a significant impairment of ET, as a measure of affective social cognition, in ALS patients with executive dysfunctions compared to healthy subjects. Probably, these inconsistent results may be due by the fact that the anatomical and biological bases of the ToM performances are still debated. In this regard, it has been suggested that ToM is a complex function involving multiple subprocesses and, thus, the evidence for a ToM network may be considered limited and contentious (Poletti et al., 2012; Schaafsma et al., 2015). Moreover, there is an increasing propensity to abandon the notion that ToM is a single cognitive ability grounded in a single set of brain regions, in favor of the potential existence of a reliably activated functional brain network (Schaafsma et al., 2015).

To note, the patients examined in the present study did not show cognitive or behavioral impairment that would negatively impact their performance in the ToM tasks proposed, except for the performance on the memory prose test. This finding corroborated previous evidence that also memory functions, although to a milder degree than executive abilities, may be affected in the ALS course (Machts et al., 2014; Raaphorst et al., 2015; Beeldman et al., 2016). In particular, the anatomical hallmarks of memory dysfunction in ALS have been recently identified in a progressive disease-related decline of hippocampal volume (Abdulla et al., 2014; Raaphorst et al., 2015). Moreover, although memory impairment may characterize cognitive profile of ALS patients with C9ORF72 mutation (Patel and Sampson, 2015), no patient studied in our work exhibited the C9ORF72 gene expansion.

Previous neuropsychological evidence, derived from cross-sectional (Abrahams et al., 1997; Montuschi et al., 2015) and longitudinal (Abrahams et al., 2005; Schreiber et al., 2005; Elamin et al., 2012) studies, identified a prominent decline of executive and language performances in ALS patients, more strikingly in bulbar (Montuschi et al., 2015) and pseudobulbar (Abrahams et al., 1997) phenotypes. In particular, some longitudinal studies identified cognitive decline early in the disease course, showing a prominent impairment of cognitive domains attributed to frontal and temporal lobes (Abrahams et al., 2005; Schreiber et al., 2005; Elamin et al., 2012). With regard to deterioration of cognitive functions across the ALS course, Schreiber et al. (2005) showed that cognitive deficits did not progress in synchrony with motor decline, but more slowly, although patients with bulbar-onset ALS had a relatively greater cognitive impairment over time than subjects with spinal-onset. More recently, Elamin et al. (2012) showed that detection of executive dysfunctions at ALS onset might be associated with significantly faster motor decline, particularly in the bulbar sites. In line with this evidence, the results of our analysis, reporting significantly lower ET scores in patients with bulbar-onset compared to those with spinal-onset, more clearly suggest that ALS patients with bulbar-onset may be also more prone to impairment of social-affective abilities than those with spinal-onset, reflecting recent findings by Burke et al. (2016a), who revealed that bulbar-onset patients performed worse than spinal-onset ones on ET. Longitudinal studies looking at changes in ToM performance over time in case of both disease onsets should be performed to investigate if difference between bulbar and spinal onset patients persists or even increases as the disease progresses.

We also revealed that EAT scores were correlated with education and “Mental Health” measures of SF-36. The former correlation may suggest a potential role of years of education as a moderator factor of ToM abilities, especially for the affective subcomponent, as also demonstrated in studies performed in healthy populations that examined ET performances in relation to some demographic variables, including years of education (Fernández-Abascal et al., 2013; Ayesa-Arriola et al., 2016). In this regard, our results, although deserving further investigation because only correlational and not causal, may support the notion that education could influence and improve social cognition, thus encouraging the use of training programs, as demonstrated in other neuropsychiatric conditions (Rocha and Queirós, 2013; Sacks et al., 2013). Moreover, at the clinical level, the growing evidence of social cognition impairment in ALS may have crucial implications for patients’ and caregivers’ training during the whole course of the disease. However, the faster trajectory of ALS progression compared to the disease course of other neurological disorders could significantly impact the clinical efficacy and application of these approaches, thus requiring an earlier intervention for effectively impacting on social cognition. On the other hand, the crucial role of interventions aimed at reducing psychological distress in caregivers of ALS patients is in line with more recent results from our research group, performed on a larger population of patients and caregivers, which revealed significant correlations between intensity of burden and caregivers’ coping strategies (unpublished data). Furthermore, the lack of correlation shown in the present work between caregiver burden and patients’ ToM scores, together with previous evidence that revealed significant associations between higher burden in carers and higher behavioral dysfunctions in patients with ALS (Chiò et al., 2010; Burke et al., 2015) and bvFTD (Brioschi Guevara et al., 2015), underlined the fact that mainly apathy and disinhibition, but also loss of empathy, more specifically explored by a more detailed behavioral assessment (Van der Hulst et al., 2015), may negatively impact caregiver burden.

In addition, the latter significant correlation observed between EAT scores and “Mental Health” dimension of SF-36 suggests that the impairment of ToM may negatively affect mainly this aspect of patients’ QoL. In agreement with this association, although demonstrated by our analysis as only “correlational” and not causal, QoL may be considered mainly dependent on psychological and existential factors in ALS (Simmons et al., 2000), thus inducing to emphasize the role of spiritual aspects and support systems in therapeutic interventions. However, “Mental Health” and “Role Emotional” domains of SF-36 have been demonstrated less impaired during the ALS course in comparison to “Physical Functioning” and “Role Physical” domains (De Groot et al., 2007), thereby depicting diverging patterns between “physical” and “mental health” domains of QoL in those patients.

Although the interesting insights discussed, our study has some limitations, principally related to the characteristics of the cohort studied (i.e., with regard to the size, the early stage of disease and the cognitive profile), the monocentric affiliation of the patients enrolled and the neuropsychological assessment performed [i.e., we did not use ALS-specific tools, such as the Edinburgh Cognitive and Behavioral ALS Screen battery (Abrahams et al., 2014), not still validated in Italian populations at the time of this study, and did not evaluate verbal fluency indices for accounting for motor disability, but only SEF (Phukan et al., 2012; Burke et al., 2016a)]. Moreover, especially in case of full battery assessment, the use of ALS-specific psychometrics should be considered. To note, a significant limitation of our analysis is the lack of working memory assessment, although working memory may have a potential confounding effect on the ToM tasks adopted. With regard to the language domain, although verbal comprehension, screened in our study by Token Test, was not impaired in both patients and HCs, it is to take into account that social communication disorders may be more specifically linked to alterations of ToM performance. In particular, autism may provide a sound model for studying the important distinction between language and communication and, thus, the effects of communication deficit on social cognition. In fact, in case of subjects with autism spectrum disorders, who associate selective impairment of ToM performance, communication deficit has been proven to lie in the use of language to affect other minds and language has been revealed important only in so far as it may be used to give evidence of own and other people’s thoughts and intentions (Frith and Happé, 1994b; Pelphrey et al., 2011).

## Conclusion

Our preliminary findings, although would need to be generalized in the context of larger samples using appropriate adjustment for executive dysfunctions, show that the ToM subcomponents are altered from the early stage of ALS and that this dysfunction may be related to the “Mental Health” of patients. Therefore, potential clinical implications derived from our study may concern the crucial role of early psychological interventions focused on deficit of both ToM subcomponents and aimed at strengthening patients’ coping strategies, interaction with caregivers and, thus, ability to engage competently therapeutic and end-of-life decisions, consequently improving patients’ QoL. Future research may stimulate the use in clinical practice of more detailed assessment of ToM and behavioral deficits.

## Author Contributions

All the authors have participated and have made substantial contributions to the approval of the final version. FT, GS, and MS contributed to the conception and design of the work; CP, CF, TF, SD, and LL contributed to the acquisition and analysis of data; FT, GS, MS, CP, AR, MM, and GT contributed to the interpretation of data for the work. FT, GS, MS, and GT drafted the work and AR, CP, CF, LL, SD, TF, and MM revised it critically for important intellectual content.

## Conflict of Interest Statement

Outside this study, FT perceived grants from Novartis, AR grants from AISLA and Allergan, MM grants from Italfarmaco and AISLA and GT fees from Biogen and Merck Serono and grants from AISLA. All authors declare that the research was conducted in the absence of any commercial or financial relationships that could be construed as a potential conflict of interest.
